# Explainable multi-view transformer framework with mutual learning for precision breast cancer pathology image classification

**DOI:** 10.3389/fonc.2025.1626785

**Published:** 2025-07-14

**Authors:** Haewon Byeon, Mahmood Alsaadi, Richa Vijay, Purshottam J. Assudani, Ashit Kumar Dutta, Monika Bansal, Pavitar Parkash Singh, Mukesh Soni, Mohammed Wasim Bhatt

**Affiliations:** ^1^ Convergence Department, Korea University of Technology and Education, Cheonan, Republic of Korea; ^2^ Department of Computer Sciences, College of Sciences, University of Al Maarif, Al Anbar, Iraq; ^3^ School of Engineering and Technology, IILM University, Greater Noida, Gautam, Uttar Pradesh, India; ^4^ School of Computer Science and Engineering, Ramdeobaba University, Nagpur, Maharashtra, India; ^5^ Department of Computer Science and Information Systems, College of Applied Sciences, AlMaarefa University, Ad Diriyah, Saudi Arabia; ^6^ Department of Computer Science, SSD Women’s Institute of Technology, Bathinda, India; ^7^ Mittal School of Business, Lovely Professional University, Phagwara, Punjab, India; ^8^ Center for Research Impact & Outcome, Chitkara University Institute of Engineering and Technology, Chitkara University, Rajpura, Punjab, India; ^9^ Model Institute of Engineering and Technology, Jammu, Jammu and Kashmir, India

**Keywords:** explainable AI, breast cancer, pathology image classification, multi-view transformer, mutual learning, MVT-OFML

## Abstract

Breast cancer remains the most prevalent cancer among women, where accurate and interpretable analysis of pathology images is vital for early diagnosis and personalized treatment planning. However, conventional single-network models fall short in balancing both performance and explainability—Convolutional Neural Networks (CNNs) lack the capacity to capture global contextual information, while Transformers are limited in modeling fine-grained local details. To overcome these challenges and contribute to the advancement of Explainable AI (XAI) in precision cancer diagnosis, this paper proposes MVT-OFML (Multi-View Transformer Online Fusion Mutual Learning), a novel and interpretable classification framework for breast cancer pathology images. MVT-OFML combines ResNet-50 for extracting detailed local features and a multi-view Transformer encoding module for capturing comprehensive global context across multiple perspectives. A key innovation is the Online Fusion Mutual Learning (OFML) mechanism, which enables bidirectional knowledge sharing between the CNN and Transformer branches by aligning both intermediate feature representations and prediction logits. This mutual learning framework enhances performance while also producing interpretable attention maps and feature-level visualizations that reveal the decision-making process of the model—promoting transparency, trust, and clinical usability. Extensive experiments on the BreakHis and BACH datasets demonstrate that MVT-OFML significantly outperforms the strongest baseline models, achieving accuracy improvements of 0.90% and 2.26%, and F_1_-score gains of 4.75% and 3.21%, respectively. By integrating complementary modeling paradigms with explainable learning strategies, MVT-OFML offers a promising AI solution for precise and interpretable breast cancer diagnosis and prognosis, supporting informed decision-making in clinical settings.

## 1 Introduction

Breast cancer has emerged as the most prevalent cancer affecting women globally (1). Computational pathology presents a promising avenue for cancer detection and personalized medicine. In current medical procedures, pathologists visually inspect Hematoxylin and Eosin (H&E) stained tissue slides to complete diagnoses (2). Due to the global rise in cancer incidences, the workload of pathologists has significantly increased, making manual examination a limiting factor in diagnostic productivity. Thus, computer-assisted pathological analysis offers an effective solution.

Classifying pathological images primarily depends on the form and layout of cell nuclei, as their morphological alterations are key indicators for determining cancer presence (3). Clinicians must integrate both localized features and broader contextual cues within images to make accurate assessments. For instance, nuclear pleomorphism and irregular patterns offer local clues, while the structure of glandular tissues provides global context. In recent years, researchers have developed various classification models. Initially, handcrafted features and traditional classifiers were utilized, heavily relying on expert annotations. Later, Convolutional Neural Networks (CNNs) (4) became popular for breast cancer pathology image classification, offering strong feature extraction abilities. The most common malignancy to strike women worldwide is breast cancer. A promising approach to personalized treatment and cancer diagnosis is computational pathology. Pathologists visually examine tissue slides stained with Haematoxylin and Eosin (H&E) to make final diagnosis during modern medical procedures. Conventional single-network models, on the other hand, struggle to balance explainability with performance—Transformers are only able to simulate fine-grained local details, while Convolutional Neural Networks (CNNs) are unable to capture global contextual information. However, CNNs struggle with capturing long-range dependencies and may overlook structural details. Recently, Transformers (5), using multi-head self-attention, have shown effectiveness in modeling global context and long-distance relationships, though they lack the inherent biases of CNNs and depend more on large training datasets. Through visual inspection and study of H&E-stained tissue slides, pathologists can diagnose a variety of disorders by examining the tissue’s general structure and cellular features. Nuclei stain blue-purple and cytoplasm/extracellular matrix stain pink in the visual framework created by the combination of hematoxylin and eosin staining, making it simple to distinguish and identify different tissue components. Few studies have explored combining the final logits and intermediate features to exploit the complementary strengths of CNNs and Transformers. To address this gap, we propose a novel model— Explainable AI Multi-View Transformer Online Fusion Mutual Learning (MVT-OFML)—which integrates CNN and Transformer branches in a dual-network design, effectively harnessing both approaches to enhance classification performance in breast cancer pathology images. The rise in cancer cases worldwide, along with improvements in diagnosis and treatment methods, has led to a major increase in pathologists’ workload. An older population, longer life expectancies, and the requirement for more thorough tissue sample analysis are some of the causes driving this. As the world’s population grows and life expectancy rises, more cancer cases occur, which in turn increases the volume of specimens that pathologists must examine.

The key contributions of this work are:

1. A dual-branch network structure combining CNN and Transformer is designed to extract complementary local and global features from pathological images, improving breast cancer pathology image classification accuracy.
2. A novel explainable AI multi-view Transformer encoding module is designed to capture global contextual features in breast cancer pathological images by integrating encoded information from different views.
3. An online fusion mutual learning method is constructed to jointly leverage the logits output layer and intermediate feature layers, deeply mining the complementarity between heterogeneous CNN and Transformer models.

Section 2, overview of prior studies, methods, findings relevant to the proposed approach, in section 3, details of model architecture, components, and techniques used for efficient implementation. In section 4, presentation and interpretation of results, metrics, comparisons, in-depth performance evaluation, in section 5, conclude of findings, contributions, limitations, and future directions of the research work.

## 2 Related work

### 2.1 Traditional methods

Early research first performed preprocessing and segmentation on pathological images, extracted features, and then completed classification. Author (6) used a gray-level co-occurrence matrix to train classifiers to identify pathological pictures that are benign and those that are malignant. The author employed a combination of multi-scale (7) regional characteristics and wavelet transform methods to identify and separate nuclei. The most popular classification system for breast cancer is based on the immunohistochemistry expression of HER2 and hormone receptors (ER, PR) as well as the histological subtype. The four primary subtypes of breast cancer identified by this method are triple-negative, HER2-positive, luminal A, and luminal B. Furthermore, a well-known pathologic categorization system is the World Health Organization’s (WHO) classification of breast cancers, which was most recently revised in 2019. Support Vector Machine (SVM) (8) was then employed for the purpose of classifying pathological images of breast cancer. Author (9) used a bivariate model in the complex wavelet domain to perform denoising and segmentation of breast tumor images. Although these methods provide good biological interpretability and perform well in specific tasks, they require manual feature design and have limited model generalization ability. In the complex wavelet domain, a bivariate model can be an effective tool for picture denoising and breast tumor segmentation. It makes it possible to analyze image data in both the frequency and spatial domains, which helps to identify tumor patches and noise while maintaining crucial features. The Complex Wavelet Transform captures both spatial and frequency information by breaking down an image into many scales and orientations. Because it can distinguish between the texture of healthy and malignant tissue, this is especially helpful for visualizing breast tumors. While the shape of glandular tissues gives global context, nuclear pleomorphism and irregular patterns provide local insights. Researchers have created a number of classification models in recent years. At first, traditional classifiers and hand-crafted features were used, with a significant reliance on expert annotations. Convolutional Neural Networks (CNNs), which have powerful feature extraction capabilities, later gained popularity for the classification of images related to breast cancer pathology.

### 2.2 CNN-based methods

CNNs use convolution and pooling operations to extract image features without requiring any prior knowledge. Various CNN architectures, such as ResNet-50 (10) and hybrid models (11), have been used in breast pathology image analysis. Author (12) designed a 3-layer CNN to identify invasive ductal carcinoma of the breast, and the results showed better performance than traditional methods. Author (13) proposed a method based on Resolution Adaptive Network (RANet) and used ADSVM (Anomaly Detection with a Support Vector Machine) for anomaly detection. To reduce reliance on medical expert annotations, Author (14) attempted to learn transferable features from weakly labelled data and achieved competitive performance in breast cancer pathological image classification. These studies have advanced research in breast cancer pathological image classification, but due to the use of convolution and pooling operations, CNNs tend to lose global contextual information in pathological images.

### 2.3 Transformer-based methods

Despite not having the inherent biases of CNNs and relying more on extensive training datasets, transformers have demonstrated efficacy in simulating global context and long-distance interactions through the use of multi-head self-attention. LN stands for layer normalization, Multi-Head Self Attention is the multi-head attention mechanism, and Multi-Layer Perceptron is the multilayer perceptron mechanism. It is made up of two linear projections divided by a nonlinear activation function. Transformers, leveraging multi-head self-attention, capture broad contextual cues in images and have seen extensive use in computer vision tasks (15). Researcher (16) utilized self-attention to emphasize localized areas and expand the receptive field at each layer. In (17), the ViT-DeiT (Vision Transformer and Data-efficient Image Transformer) was introduced to classify breast cancer histopathology images. A developing strategy for tumor treatment and prevention in oncology is called personalized medicine (PM), often known as precision medicine. It considers the lifestyle, morbidities, and genetic and intra-tumor variability of each cancer patient. Individualized cancer care considers the variations among cancer instances and people. This can lower the chance of adverse effects and boost the effectiveness of treatment. Both the prognosis and quality of life of cancer patients can be enhanced by personalized cancer treatment. Study (18) proposed DCET-Net (Dual-stream Convolution Expanded Transformer Network), a dual-pathway model that jointly extracts detailed and holistic features for breast cancer pathology image classification. However, due to the absence of inductive bias, Transformers often struggle with local feature extraction. Researcher (19) enhanced the Swin Transformer to perform multi-class classification of breast cancer, surpassing the standard ViT. However, these studies rarely consider jointly leveraging the logits layer and intermediate feature layers to mine the complementary information between heterogeneous CNN and Transformer models. Swin Transformers typically perform better than traditional Vision Transformers (ViTs) when it comes to the multi-class classification of breast cancer. Compared to ViTs, Swin Transformers are able to capture more context and long-range dependencies because they employ a shifting window technique for self-attention. As a result, the classification of various subtypes of breast cancer is more accurate and sensitive. Swin Transformers’ shifting window technique makes it possible to better capture long-range dependencies in the image, which is essential for challenging classification jobs.

### 2.4 Multiple instance learning methods

Multiple Instance Learning (MIL) transforms the problem of pathology image classification into a weakly supervised MIL problem by dividing pathological images into instances and grouping them into bags, thereby reducing the model’s dependence on annotations. MIL performs well in handling high-resolution histopathological images and is often used for pathological diagnosis of Whole Slide Images (WSI). Author (20) proposed a weakly supervised learning framework, using a Multiple Instance Neural Network (MINN) to classify breast cancer histopathological images. Author (21) proposed an attention-based aggregation operation by incorporating a single attention module into MINN to learn additional contribution information from each instance, achieving pathology image classification. Author (22) proposed a Transformer-driven Multiple Instance Learning (TransMIL) architecture to capture relationships among various instances and categorize high-resolution histopathology images. Similarly, Author (16) introduced the explainable AI Multi-View Attention-guided Multiple Instance Detection Network (MVAMIDN) aimed at identifying breast cancer in such high-detail medical images. However, current MIL methods are unable to precisely filter instances, which limits the improvement of model performance.

In summary, deep learning models such as CNN and Transformer have played important roles for the purpose of classifying pathological images of breast cancer. Having said that, pathological picture classification for breast cancer relies not only on local nuclear features but also on the global contextual information of breast tissue structure. Existing work has not fully leveraged the respective strengths of CNN and Transformer. CNNs can be combined with other architectures, such as RNNs or Transformers, to take advantage of their distinct advantages for tasks like medical picture segmentation and image recognition. For instance, whereas RNNs are able to capture sequential dependencies, CNNs are particularly good at extracting spatial information. Utilizing the last logits and intermediate characteristics, CNNs and Transformers can capitalize on their complementing strengths. Our proposal, Explainable AI Multi-View Transformer Online Fusion Mutual Learning (MVT-OFML), bridges this gap by combining CNN and Transformer branches into a dual-network architecture. To address this, a dual-branch network structure combining CNN and Transformer is designed, where ResNet-50 is used to identify specific characteristics among breast cancer pathology pictures; as well as With the help of the transformer encoding module, pathological pictures of breast cancer can have their global contextual information extracted more effectively; and an online fusion mutual learning method is constructed to build a mutual learning channel between ResNet-50 and Transformer. Specifically, an ensemble classifier and a fusion classifier are designed to jointly use the logits output layer and intermediate feature layers to achieve mutual learning between models, deeply mining the correlation between heterogeneous CNN and Transformer models to accomplish breast cancer pathology image classification.

## 3 Model implementation

**Figure 1:**
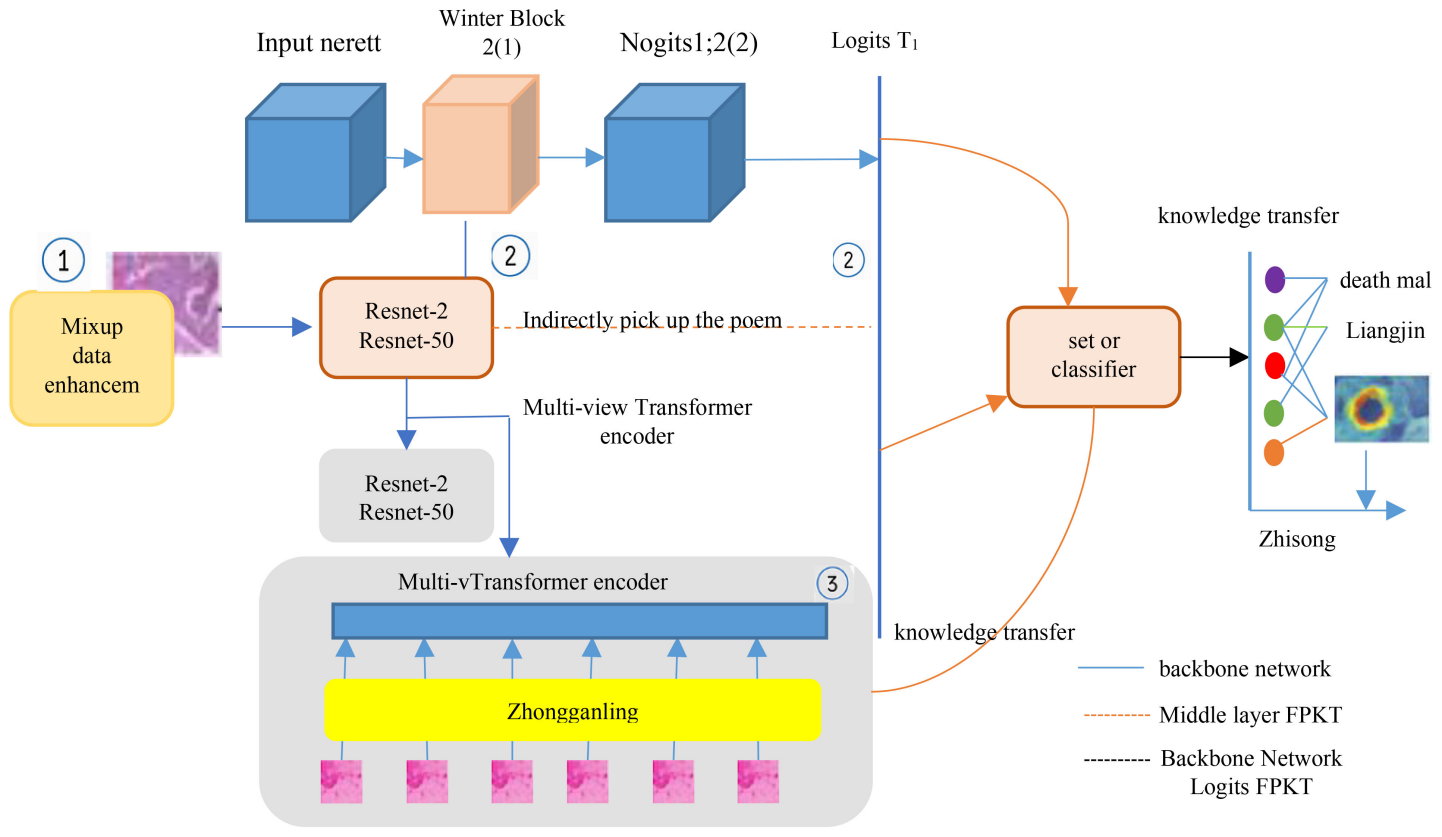
MVT-OFML model overview.

The MVT-OFML model is shown in **Figure 1**. This model is an end-to-end dual-branch network, consisting of a CNN backbone network and a Transformer backbone network.

The model executes the following steps:

Phase 1: Mixup (19) is used to obtain a sufficient number of high-quality training images. Mixup uses linear interpolation of related labels during augmentation to expand the training distribution, generating high-quality image samples while maximally retaining the important information from the original pathological images.
Phase 2: A dual-branch network structure composed of CNN and Transformer is constructed. A multi-view Transformer encoder is designed to better extract and fuse global contextual features in the image; ResNet-50 is selected to capture local features in the image. An ensemble classifier is designed to integrate logits from the two heterogeneous networks, preparing for the subsequent stimulation of the fusion classifier.
Phase 3: An online fusion mutual learning method is designed. The third convolutional layer of ResNet-50 is selected as Feature Map 1, and the third layer of the multi-view Transformer is selected as Feature Map 2. Feature Map 1 and Feature Map 2 are combined as intermediate features, which are used as input for the fusion classifier. The fusion classifier adaptively fuses the intermediate features from the two backbone networks to fully exploit the implicit complementarity between the heterogeneous networks.

**Figure 2:**
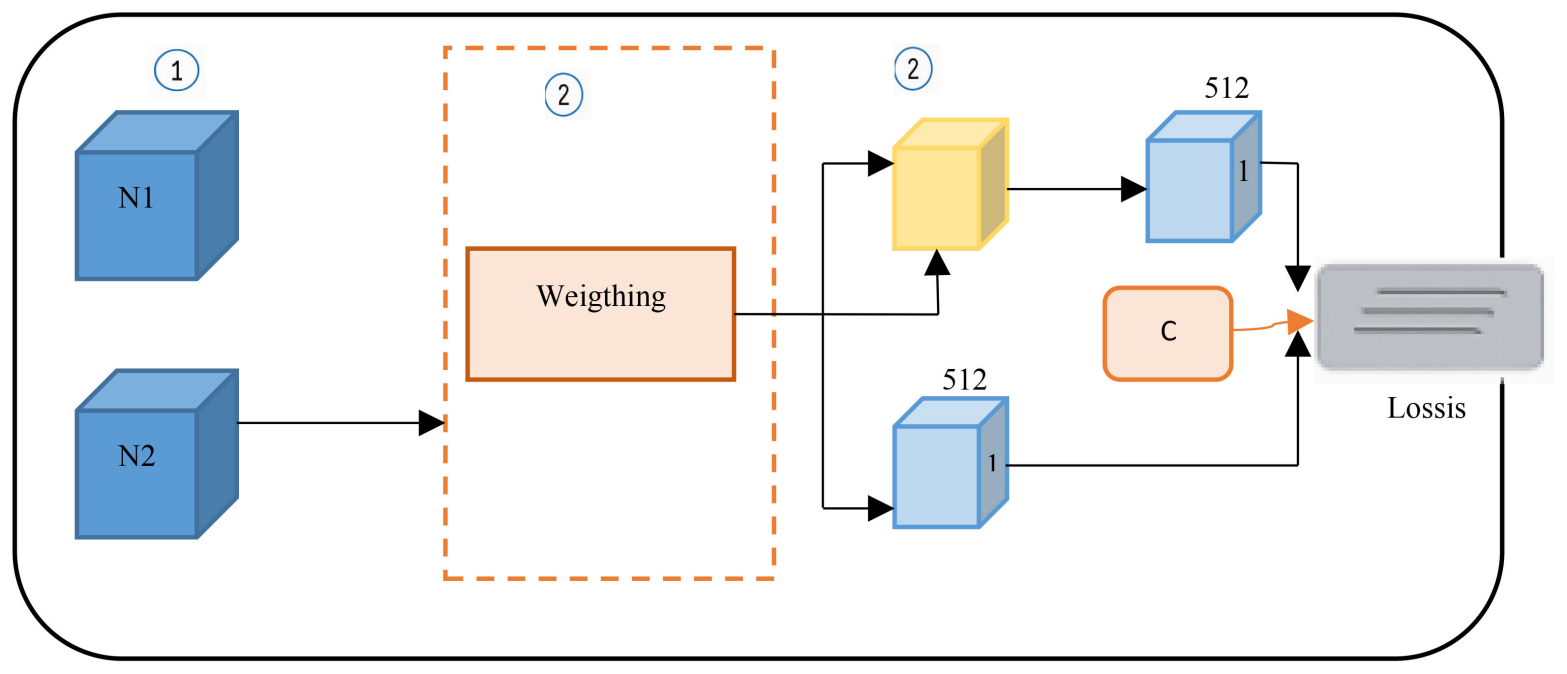
Fusion classifier.

As shown in **Figure 2**, the fusion classifier first applies adaptive average pooling to Feature Map 1 (512, 28, 28) and Feature Map 2 (512, 14, 14). After adaptive pooling, the width and height of each feature map are reduced to 1; subsequently, concatenation is applied to complete the feature fusion. The total number of channels in the combined feature map equals the sum of the channels from the contributing feature maps, i.e., 1024. A pointwise convolution is then performed to adjust the number of channels in the merged feature map to C (where C corresponds to the number of classes in the dataset), improving the abstract representation capacity of the local module. Adaptive average pooling and pointwise convolution not only ensure that the fusion classifier can adaptively match any two feature maps but also avoid the need for a large number of tunable parameters, which is a drawback of traditional fusion methods. At the same time, the output from the ensemble classifier is passed into the fusion classifier—i.e., the logits-level information is used to stimulate the fusion classifier. Then, the information in the fusion classifier is passed back to the two backbone networks, i.e., by the utilization of the intermediate a layer for features and another for logits output in tandem to further enhance pathological knowledge exchange between the backbone networks. Through mutual learning, the performance of each backbone network is improved.

Finally, the classification is obtained by calculating the fusion classifier’s categorization likelihood result of breast cancer pathological images.

### 3.1 Multi-view transformer encoding

**Figure 3:**
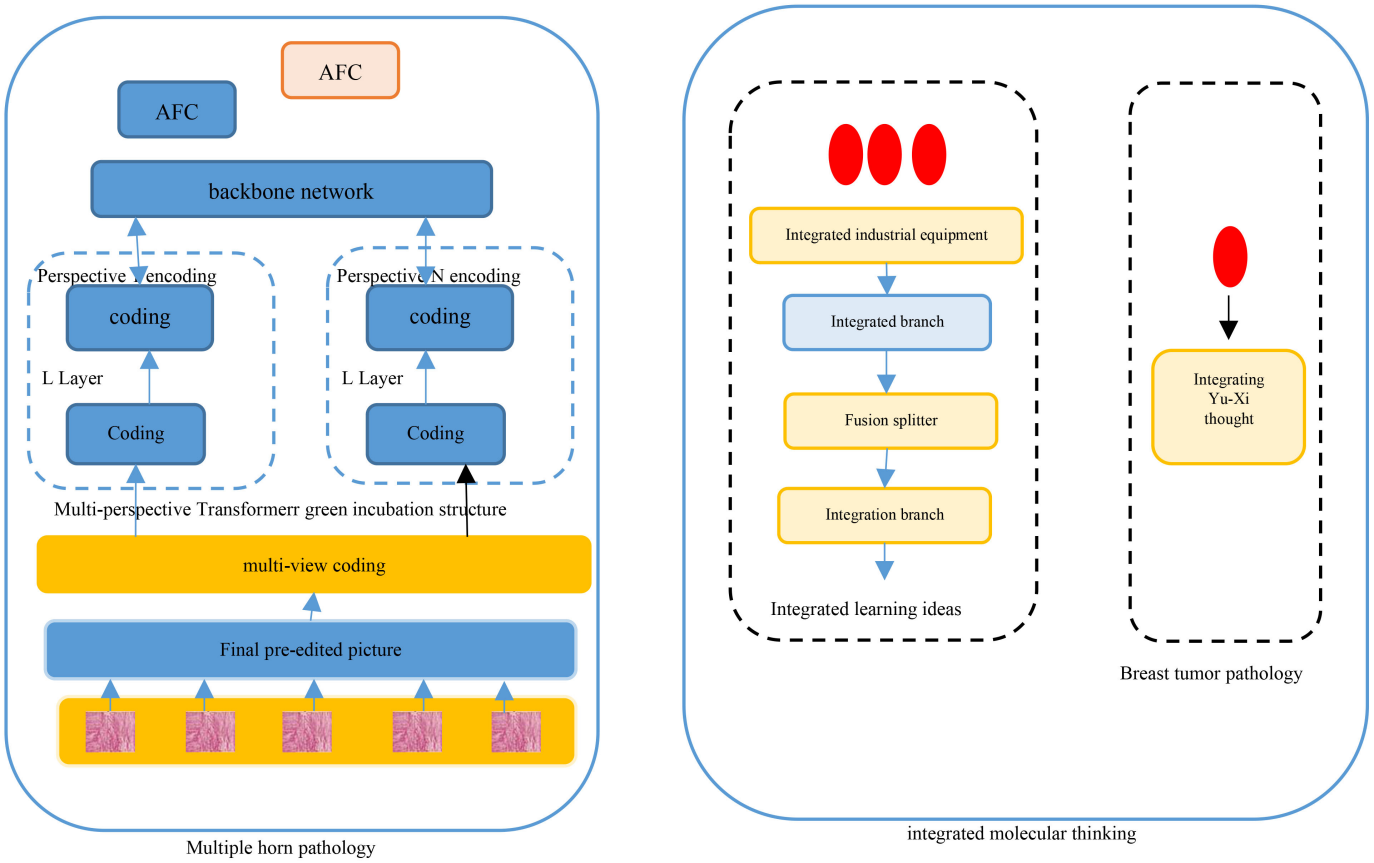
Details of the explainable AI multi-view transformer encoding module implementation.

The traditional Transformer network does not adequately capture the complex textures, structures, and spatial information in breast cancer pathological images. Multiple-view explainable AI The transformer encoding module is intended to provide global contextual information and a more accurate description of the structural characteristics of breast cancer tissue. Multi-view fusion and global encoder modules are part of the explainable AI multi-view Transformer encoder. In order to manage challenging multi-classification jobs and attain superior performance, the explainable AI multi-view Transformer encoder can extract more reliable and efficient features regardless of magnification, learning important information from other problematic categories. Therefore, a explainable AI multi-view Transformer encoding module is designed to better characterize the structural features of breast cancer tissue and global contextual information. The explainable AI multi-view Transformer encoding module is shown in **Figure 3**.

First, the input breast cancer pathological image is represented as $x\in {\mathbb{R}}^{\mu \times Z\times C}$ , where H represents the image height, Z represents the image width, and C represents the image channels. After cropping, the image is divided into N=(H×Z)/Q image patches of size Q×Q×C , where Q is the height and width of the cropped image patch. These image patches are then flattened into one-dimensional vectors $x_{\text{patch }}\in {\mathbb{R}}^{N\left(Q\times Q\times C\right)}$ . After a linear mapping, these one-dimensional vectors produce a sequence of token embeddings $A^{0}$ containing pathological feature information. This sequence is prefixed with a learnable class token $A_{\text{class }}=A^{d}$ and a positional embedding $q\in {\mathbb{R}}^{\left(N+1\right)\cdot d}$ , where d is the dimension of the input embedding vector and E denotes the matrix implementing the linear mapping. Thus, the token embedding sequence is as shown in Equation 1:


(1) $$A^{0}=\left[\begin{array}{lllll} A_{\text{class }} & x_{\text{patch }}^{1}E & x_{\text{patch }}^{2}E & \cdots & x_{\text{patch }}^{N}E \end{array}\right]+q$$

The token embedding sequence $A^{0}$ is processed by N view-specific Transformer encoders, each encoder containing L layers arranged sequentially. The explainable AI multi-view Transformer encoder includes a multi-view fusion module and a global encoder module. As shown in **Figure 3**, the explainable AI multi-view fusion module integrates encoded information from other views and performs multidimensional interactive processing of token sequences through Cross View Attention (CVA) to extract the interacted pathological information. CVA is formulated as shown in Equation 2:


(2) $$\text{CVA}\left(x,y\right)=\text{Softmax}\left(\frac{Z^{Q}xZ^{K}y^{\text{T}}}{\sqrt{d_{k}}}\right)Z^{V}y$$

Here, $Z^{\varrho },Z^{\kappa }$ and $Z^{V}$ are three vector matrices obtained by projecting the sequence through mapping matrices in the self-attention layer; y represents a learnable parameter matrix. Based on CVA, $A^{\left(i\right)}$ represents the features of the sequence from the **i** view encoder, $A^{\left(i+1\right)}$ represents the feature information of the sequence from the (i+1) view encoder, and $Z^{qmj}$ denotes the projection vector. *A^i^* is formulated as shown in Equation 3:


(3) $$A^{i}=\text{CVA}\left(A^{\left(i\right)},Z^{\text{proj }}A^{\left(i+1\right)}\right)$$

CVA ranks and integrates the features output from two adjacent views based on the importance of pathological features to obtain richer pathological features. Since the hidden dimensions between different views are different, it is necessary to project the view features of different dimensions to the same dimension, and then fuse the two after projection. Finally, the token information after explainable AI multi-view encoding and interaction fusion is input into the global encoder. The explainable AI Multi-View Attention-guided Multiple Instance Detection Network is demonstrated in medical photographs. The diagnosis of breast cancer from histological images has advanced significantly thanks to deep learning techniques. It is still difficult to train an interpretable diagnosis algorithm with high-resolution histopathology images. A key field of study to tackle the interpretability issues presented by intricate machine learning models is Explainable Artificial Intelligence (XAI). The computations of the global encoder and multilayer perceptron are shown in Equations 4 and 5:


(4) $$y^{l}=\text{MSA}\left(\text{LN}\left(A^{l-1}\right)\right)+A^{l-1}$$

(5) $$A^{l}=\text{MLP}\left(\text{LN}\left(y^{l}\right)\right)+y^{l}$$

MSA (Multi-Head Self Attention) is the multi-head attention mechanism (6), LN represents layer normalization (6), and MLP (Multi-Layer Perceptron) is the multilayer perceptron mechanism, this is comprised of a nonlinear activation function that divides two linear projections GELU (Gaussian Error Linear Units). The final classification information is obtained from the multilayer perceptron.

### 3.2 Online fusion mutual learning

In offline Knowledge Distillation (KD), the student network’s performance deteriorates as the chasm between student and teacher networks’ capacities grows. Online KD has many benefits over its offline counterpart: links between educators and their students can simultaneously perform the distillation operation. Few studies jointly exploit complementary information between heterogeneous CNN and Transformer models by combining both the logits layer and intermediate feature layers. MVT-OFML (Multi-View Transformer - Online Fusion Mutual Learning) combines the output of the logits layer of both networks combined with the combined data from different feature maps, fully mining the pathological knowledge between heterogeneous networks to jointly supervise network training, ultimately establishing a connection where the two diverse networks can learn from each other. The field and application determine how well global contextual modelling, or global context modelling, works. In disciplines like computer science, it refers to the process of generating predictions or hypotheses based on data from several locations or domains, which may result in effective algorithms. In other fields, such as psychology, the focus is on comprehending how perceptions about one’s own talents and self-efficacy are influenced by external events. In online fusion learning, two heterogeneous backbone networks are used to carry out mutual learning. In this work, ResNet-50 is used as Backbone Network 1, and the explainable AI multi-view encoding Transformer from Section 3.1 is used as Backbone Network 2. Let the N pathological image samples in m classes $X=\left\{x_{i}\right\}$ , with their corresponding labels $Y=\left\{y_{i}\right\}$ , where $y_{i}\in \left\{1,2,\cdots ,m\right\},i=1,2,3,\cdots ,N$ . The softened probabilities for the m classes of sample $x_{i}$ output by ResNet-50 and the explainable AI multi-view encoding Transformer are defined by Equations 6 and 7, respectively.


(6) $$q_{1}^{m}\left(x_{i},T\right)=\frac{\text{exp}\left(A_{1}^{m}/T\right)}{\sum_{m=1}^{M}\text{exp}\left(A_{1}^{m}/T\right)}$$

(7) $$q_{2}^{m}\left(x_{i},T\right)=\frac{\text{exp}\left(A_{2}^{m}/T\right)}{\sum_{m=1}^{M}\text{exp}\left(A_{2}^{m}/T\right)}$$

Here, $A_{1}^{m}$ is the feature from the Softmax layer of ResNet-50, $A_{2}^{m}$ is the feature from the MLP layer of the explainable AI multi-view Transformer, Where T stands for the temperature at which distillation takes place. A more uniform distribution of problematic classes is associated with a bigger value of T. Consequently, the distillation temperature now more effectively softens the probabilities, allowing for the extraction of more relevant pathological information from linked pathological categories. This, in turn, improves the accuracy of breast cancer pathological picture classification.

Due to the complexity of pathological images, the embedded fusion mutual learning framework also includes an ensemble classifier, which is used to gain more useful pathogenic understanding from both backbone subnetworks. As shown in **Figure 1**, the logits output of the ensemble classifier is calculated as shown in Equation 8:


(8) $$A_{e}=\frac{A_{1}+A_{2}}{2}$$

The ensemble’s cross-entropy loss classifier is calculated as shown in Equation 9:


(9) $$L_{\text{ensemble }}^{f}=\sum_{i=1}^{N}\sum_{m=1}^{M}I\left(y_{i},m\right)\text{log}\left(q_{e}^{m}\left(x_{i},1\right)\right)$$

The ensemble classifier feeds the fusion classifier pathological knowledge during each training iteration, drawing from ResNet-50 and the explainable AI multi-view Transformer encoder. To determine the loss of this Ensemble Pathological Knowledge Transfer (EPKT) mechanism, which is based on KL divergence, one follows these steps as shown in Equation 10:


(10) $$L_{\text{EPKT}}=D_{\text{KL}}\left(q_{\epsilon }∥q_{f}\right)$$

For the multi-classification task, the objective loss function of ResNet-50 is defined as the cross-entropy loss $L_{\text{nett }}^{1}$ between the predicted labels and the true labels. In the online fusion mutual learning framework, the combined pathology expertise of the two primary networks and the fusion classifier are transferred to each other, promoting performance improvement of both the backbone networks and the fusion classifier. The posterior probability $q_{f}$ of the fusion classifier provides training experience, and the KL divergence is used to quantify the difference between $q_{1}$ and $q_{f}$ (i.e., $D_{\text{KL}}\left(q_{1}∥q_{f}\right))$ . The specific formula is as shown in Equation 11:


(11) $$\begin{array}{c} L_{net1}^{1}=\sum_{i=1}^{N}\sum_{m=1}^{M}I\left(y_{i},m\right)\log\left(q_{1}^{m}\left(x_{i},1\right)\right)\\ L_{FPKT}^{1}=D_{KL}\left(q_{1}\|q_{f}\right)=\sum_{i=1}^{N}\sum_{m=1}^{M}q_{e}^{m}\left(x_{i}\right)\log\frac{q_{f}^{m}\left(x_{i}\right)}{q_{1}^{m}\left(x_{i}\right)}\\ L_{1}=L_{\text{net}1}^{1}+T^{2}\times L_{\text{FPKT}}^{1} \end{array}$$

Here, I symbolize the following indicator function shown in Equation 12:


(12) $$I\left(y_{i},m\right)=\{\begin{array}{l} 1,y_{i}=m\\ 0,y_{i}\neq m \end{array}$$

$L_{1}$ is the total loss function of ResNet-50, which consists of ResNet-50’s $L_{\text{net}1}^{1}$ loss and the fusion classifier’s $L_{\text{EPKT}}$ loss. The $L_{\text{EPKT}}$ loss encourages the network so that better feature maps may be generated and fusion performance can be enhanced. Each backbone network receives its pathological knowledge from the fusion branch, which is integrated in the intermediary feature layers. Fusion Pathological Knowledge Transfer is the process that each backbone network goes through to improve its training, which transfers the softened probability distribution of the fusion classifier to each backbone network. The combination of the backbone network logits output and the pathological knowledge from the fusion classifier improves the final performance.

Similarly, the total loss function of the explainable AI multi-view Transformer encoder is expressed as Equation 13:


(13) $$\begin{array}{c} L_{\text{net}2}^{2}=\sum_{i=1}^{N}\sum_{m=1}^{M}I\left(y_{i},m\right)\text{log}\left(q_{2}^{m}\left(x_{i},\text{l}\right)\right)\\ L_{FPKT}^{2}=D_{KL}\left(q_{2}\|q_{f}\right)=\sum_{i=1}^{N}\sum_{m=1}^{M}q_{e}^{m}\left(x_{i}\right)\log\frac{q_{f}^{m}\left(x_{i}\right)}{q_{2}^{m}\left(x_{i}\right)}\\ L_{2}=L_{\text{net}2}^{2}+\;T^{2}\times L_{\text{FPKT}}^{2} \end{array}$$

According to Equation 13, T_2_ is multiplied due to the loss of transferred pathogenic knowledge caused by the matching merged softened probability distribution is scaled by $T^{2}$ . Finally, the overall loss of the model is formulated as shown in Equation 14:


(14) $$L_{f}=L_{1}+L_{2}+T^{2}\times L_{\text{ensemble }}^{f}$$

Training for the fusion module and the two main networks happens at the same time. Every mini-batch update incorporates and maintains a collaborative learning strategy, phase, extracting supervisory cues from the annotations. Simultaneously, the fusion module transfers the combined pathological insights back to each backbone network, encouraging the ResNet-50 and explainable AI multi-view Transformer encoder to capture richer pathological features from diverse intermediate representations. The goal of Explainable Artificial Intelligence (XAI) is to decipher the “Black Box,” inspire confidence, and make it easier to incorporate AI into clinical decision-making processes for the best possible outcomes. Transformer Online Fusion Mutual Learning), a new and comprehensible pathological picture categorization system for breast cancer. A multi-view Transformer encoding module is used in MVT-OFML to capture full global context from several views, while ResNet-50 is used to extract detailed local features. Additionally, the ensemble classifier relays the deep pathological information from each backbone to the fusion module, promoting its training. Consequently, both the logits and distilled features from the backbones enhance the fusion classifier’s discriminative power. The backbone networks and fusion classifier reinforce and complement one another in order to glean extensive information for the interpretation of diseased images.

## 4 Experimental results and detailed analysis

### 4.1 Datasets

This paper uses two public datasets to verify the effectiveness and robustness of the model. The details of the datasets are as follows:

1. BreakHis: It comprises four magnification settings — 40x, 100x, 200x, and 400x. Each breast tissue pathology image contains 3 RGB channels, with each channel having an 8-bit depth. The resolution of each image is 700×460 pixels. By identifying significant variations in their form and growth patterns, such as boundary definition, the existence of soft tissue mass, and cortical involvement, pathological pictures of benign and malignant tumors can be differentiated. In contrast to malignant tumors, which frequently have ill-defined boundaries, rupture the cortex, and may be accompanied by soft tissue masses, benign tumors typically have well-defined borders, do not break the cortex, and may not be linked with soft tissue masses. The BreakHis dataset contains 2,408 benign and 5,429 malignant tumor samples, categorized into eight subtypes: adenosis (A), fibroadenoma (F), phyllodes tumor (PT), tubular adenoma (TA), ductal carcinoma (DC), lobular carcinoma (LC), mucinous carcinoma (MC), and papillary carcinoma (PC). This study performs binary and 8-class classification tasks using the BreakHis dataset. The Mixup technique expands the dataset by a factor of four to help mitigate overfitting. **Table 1** presents the dataset’s distribution after Mixup augmentation.
2. BACH: BACH classifies microscopic images into four categories: Normal, Benign, *In situ* carcinoma, and Invasive carcinoma. The dataset contains 400 RGB images, each 2048×1536 pixels, with 0.42 μm per pixel resolution in **Table 2**.

**Table 1:** Distribution of the BreakHis dataset after Mixup data augmentation.

Dataset	Training		Testing	
	Benign images	Malignant images	Benign images	Malignant images
Original dataset	1 736	3 800	744	1 629
Enhanced dataset	6 944	15 200	744	1 629

**Table 2:** Distribution of the BACH dataset after mixup data augmentation.

Dataset	Training				Test			
	Normal	Benign	Carcinoma in situ	Invasive carcinoma	Normal	Benign	Carcinoma in situ	Invasive carcinoma
Original dataset	70	70	70	70	30	30	30	30
Enhanced dataset	280	280	280	280	30	30	30	30

In summary, the two datasets have significant differences in terms of quantity, resolution, etc. Conducting experiments on them helps better verify the robustness of the MVT-OFML model.

### 4.2 Deployment info

The model proposed in this study is developed using PyTorch, with all experiments carried out on a high-performance machine equipped with dual GTX 3080Ti GPUs. To ensure effective training, the Adam optimizer is employed, initialized learning rate 0.01, momentum 0.8, and weight decay 5×10^-4^. The temperature parameter T in the loss formulation is fixed at 4. A trainable positional embedding is included in the setup. Following Mixup augmentation, images are resized to 224×224 before being passed into the network. A batch size of 64 is used, and training spans 300 epochs. The dataset is divided with a 70:30 split between training and testing. For thorough evaluation, five performance indicators are applied: accuracy, AUC, precision, recall, and F1-score.

### 4.3 Data from the experiment

#### 4.3.1 Evidence from experiments conducted on the BreakHis database

First, the MVT-OFML model is applied to Using the BreakHis dataset, we performed binary classification and 8-class classification tasks, and we compared the results to those of popular breast cancer pathology picture classification models. Various models and methodologies are evaluated using four metrics: accuracy, precision, recall, and F1-score. The detailed comparison results for binary classification are shown in **Table 3** (bold data indicates the best results).

**Table 3:** Comparison of binary classification performance between the MVT-OFML model and mainstream methods on the BreakHis dataset.

Backbone network or method	Year of publication	Magnification/times	Accuracy/%	Accuracy/%	Recall rate/%	F_1_ score/%
IDSNet (20)	2020	40	91.5	90.55	91	90.54
		100	90.4	91.23	90.56	90.78
		200	95.3	95.33	95.66	95.39
		400	86.7	89.35	88.42	89.47
DCET-Net (18)	2021	40	99	99.47	97.38	98.41
		100	98.08	94.79	98.91	96.81
		200	99.34	97.66	97.82	98.82
		400	98.72	98.22	97.65	97.93
RANet-ADSVM (13)	2022	40	91.96	93.83	94.91	94.36
		100	96.83	98.52	98.3	98.32
		200	98.05	98.92	99.15	99.13
		400	90.3	93.17	93.56	93.35
VIT-DeiT (17)	2022	40	99.43	99.38	99.46	99.4
		100	98.34	98.31	98.51	98.35
		200	98.27	98.32	98.27	98.23
		400	98.82	98.57	98.78	98.65
MVT-OFML	2023	40	99.77	99.75	99.71	99.77
		100	99.56	99.74	99.54	99.44
		200	99.76	99.65	99.43	99.62
		400	99.45	99.3	99.69	99.33
Improvement		40	0.34↑	0.37↑	0.25↑	0.37↑
		100	1.22↑	1.43↑	1.03↑	1.09↑
		200	1.40↑	1.33↑	1.16↑	1.39↑
		400	0.63↑	0.73↑	0.96↑	0.91↑

The symbol “↑”defines the improved values.

From **Table 3**, it can be seen that the MVT-OFML model achieved the best accuracy of 99.77% in the 40x binary classification task. Compared with the strongest model ViT-DeiT, the MVT-OFML model achieved optimal performance in all four metrics. From the improvement, it can be seen that the MVT-OFML model outperforms the best baseline by 0.25% to 1.53%, indicating that the MVT-OFML model can effectively improve breast cancer pathology image classification performance. Depending on the approach taken, different breast cancer pathology pictures have different levels of accuracy, particularly when it comes to differentiating between benign and malignant cases. Studies reveal that deep learning models can classify histopathology pictures with high accuracy, frequently outperforming human pathologists, even though manual examination by pathologists is still the gold standard. Under a microscope, pathologists are taught to identify minute morphological changes in cells and tissues in order to diagnose breast cancer. They are the gold standard, but depending on experience, skill, and case complexity, their accuracy may differ. More importantly, under different magnification levels, the MVT-OFML model achieves more balanced performance, thanks to the explainable AI multi-view Transformer encoder’s ability to extract more robust and effective features independently of magnification levels, ultimately improving classification performance. In terms of the average accuracy across the four magnifications, IDSNet, DCET-Net, RANet-ADSVM, and ViT-DeiT models achieved average accuracies of 90.98%, 98.79%, 94.29%, and 98.72%, respectively, in contrast to the MVT-OFML model, which averaged 99.64% accuracy. Other measures (accuracy, recall, and F1-score) likewise showed that the MVT-OFML model was the most stable and resilient, and it also attained the best average performance.

At 40x magnification, the MVT-OFML model outperformed the single-stream model IDSNet in terms of accuracy (8.27 percent), precision (9.28 percent), recall (8.71 percent), and F1-score (9.23 percent). This occurs because the MVT-OFML model makes excellent use of the complementing pathological information present in various networks, under the dual-stream network architecture. The form and arrangement of cell nuclei are the primary determinants of pathological image classification because these morphological changes are important markers for identifying the existence of malignancy. To accurately analyze photos, clinicians must incorporate both localized features and more general contextual clues. Nuclear pleomorphism and irregular patterns, for example, provide local indications, whereas glandular tissue structure gives a global context. Numerous classification models have been created by scholars in recent years. Compared with the dual-stream model ViT-DeiT, MVT-OFML improved the accuracy, precision, recall, and F1-score at 400x magnification by 0.63%, 0.73%, 0.91%, and 0.68%, respectively. ViT-DeiT only utilizes the final logits output, while the pathological features hidden in the intermediate layers are not fully exploited. In contrast, MVT-OFML bridges the “gap” between heterogeneous CNN and Transformer models. It jointly mines complementary information between heterogeneous CNN and Transformer models by deeply utilizing both the logits layer and the intermediate feature layers, fully harnessing their advantages to extract more discriminative pathological image features. In summary, compared with various baseline methods, the approach suggested in this article — MVT-OFML — is optimal.

**Figure 4:**
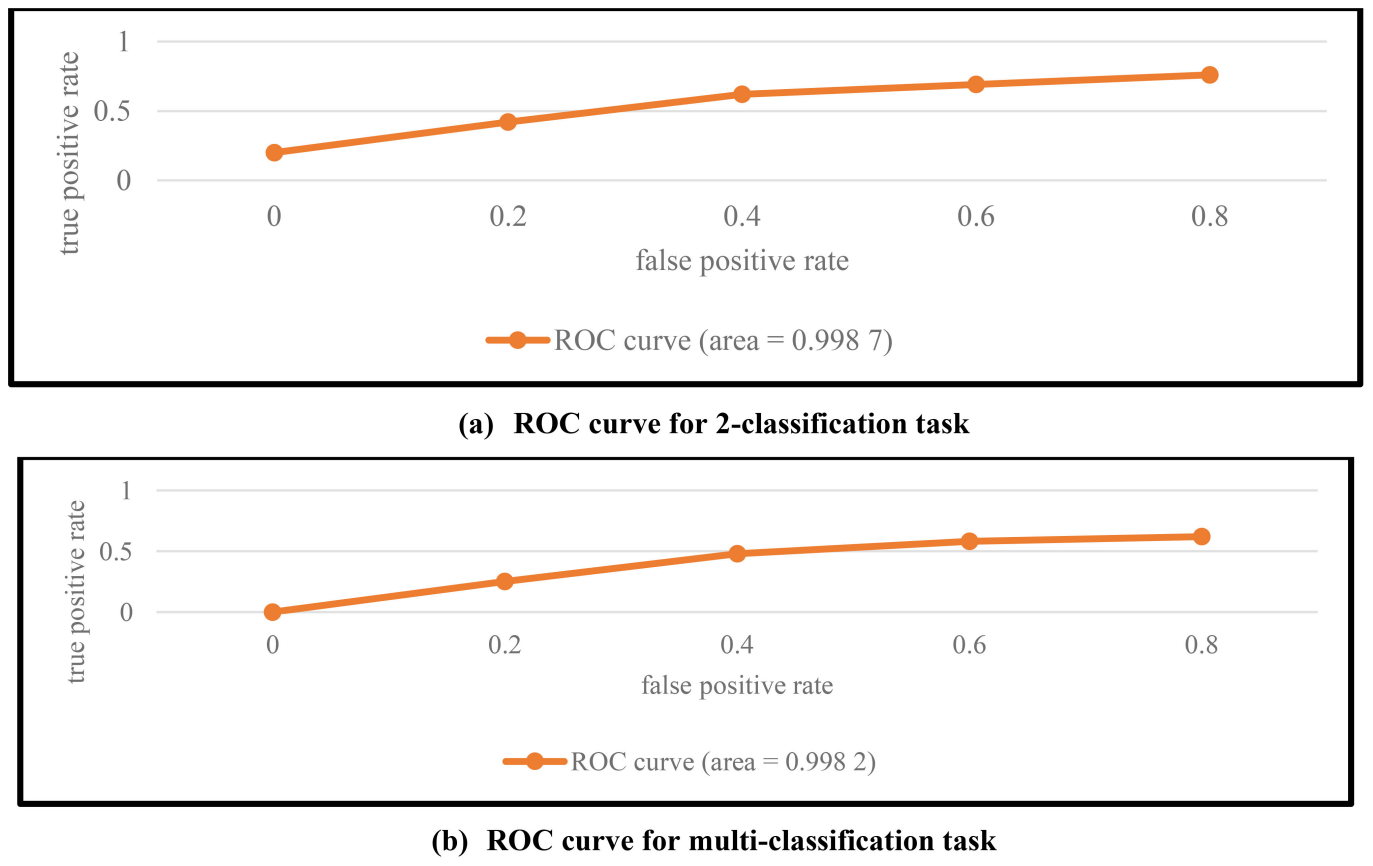
ROC curves of the MVT-OFML model on the BreakHis dataset.

**Figure 4a** also displays the BreakHis dataset’s binary classification ROC curve. The MVT-OFML model’s ROC curve, as seen in **Figure 4a**, is significantly closer to the point (0,1) than it is to the 45° diagonal line of the coordinate axes. The model’s AUC reaches 0.9987. The excellent ROC curve clearly demonstrates that the MVT-OFML model fully exploits the advantages of CNN and Transformer models in their respective feature learning, and the complementary pathological information between heterogeneous models extracts more discriminative pathological image features to improve various performance metrics of the model, making it efficient and practical in regard to the binary classification assignment involving photos of breast cancer pathology. Overall, the MVT-OFML model achieved good performance under four different magnification levels and across various performance evaluation metrics, further proving that the MVT-OFML model has good generalization ability and strong robustness. At the same time, it also shows that the MVT-OFML model is effective in the context of pathology image classification in breast cancer. Photography should use both precise localized details and more general contextual clues to guarantee fair and pertinent assessments. This method helps steer clear of biased judgments and offers a deeper comprehension of the scene. Contextual cues help viewers comprehend the importance of localized elements in relation to the overall picture. Contextual cues help viewers comprehend the importance of localized elements in relation to the overall picture.

Breast cancer histological picture multi-class categorization is more difficult than binary classification. **Table 4** displays the exact quantitative results of an 8-category classification task that was conducted on the BreakHis dataset using the MVT-OFML model. The top performing models are indicated by bold figures. The four measures that were used for evaluation were F1-score, recall, accuracy, and precision. **Figure 4b** shows the comparable 8-class ROC curve on the BreakHis dataset.

**Table 4:** Comparison of classification performance between the MVT-OFML model and other mainstream methods on the BreakHis dataset.

Backbone network or method	Year of publication	Magnification/times	Accuracy/%	Accuracy/%	Recall rate/%	F_1_ score/%
Deep-Net (11)	2020	40	94.43	95.25	95.55	95.39
		100	94.45	94.64	94.64	94.42
		200	92.27	90.71	92.24	91.42
		400	91.15	90.74	91.09	90.75
AnoGAN (21)	2021	40	99.15	99.64	99.46	99.78
		100	97.09	98.07	98.49	98.22
		200	87.58	88. 19	92.82	90.62
		400	87.3	82.77	92.5	88.23
BHC-Net (22)	2022	40	94.71	95.25	95.55	95.39
		100	94.6	94.51	94.64	94.42
		200	92.35	90.71	92.24	91.42
		400	91.5	90.74	91.09	90.75
BreaST-Net (23)	2022	40	96	—	—	95.8
		100	92.6	—	—	92.4
		200	93.5	—	—	93.6
		400	91.5	—	98.88	93.2
MVT-OFML	2023	40	99.19	98.93	98.9	98.46
		100	99.05	97.44	99.3	97.77
		200	99.6	97.88	99.54	99.33
		400	99.63	96.19	—	98.45
Improvement		40	3.19↑	—	—	2.66↑
		100	6.45↑	—	—	5.37↑
		200	6.10↑	—	—	5.73↑
		400	8.31↑	—		5.25↑

The symbol “↑”defines the improved values.

**Table 4** shows that at any magnification level, the MVT-OFML model outperformed the best baseline in the literature, BreAST-Net, in terms of classification performance, with all metrics reaching saturation. As an example, the MVT-OFML model attained the highest accuracy of 99.63% in the 8-class classification assignment conducted at 400x magnification. Similarly, in terms of the average accuracy across four magnifications, the Deep-Net (11), AnoGAN (21) (Anomaly detection with Generative Adversarial Networks), BHC-Net (22), and BreaST-Net (23) models achieved average accuracies of 93.08%, 92.78%, 93.29%, and 93.40%, respectively, while the MVT-OFML model achieved the best average accuracy of 99.36%. Moreover, the average performance of other metrics (precision, recall, and F1-score) was also best with MVT-OFML. This indicates that whether in terms of the best results or average performance, the MVT-OFML model is optimal, strongly proving the model’s overall robustness and reliability.

**Figure 5:**
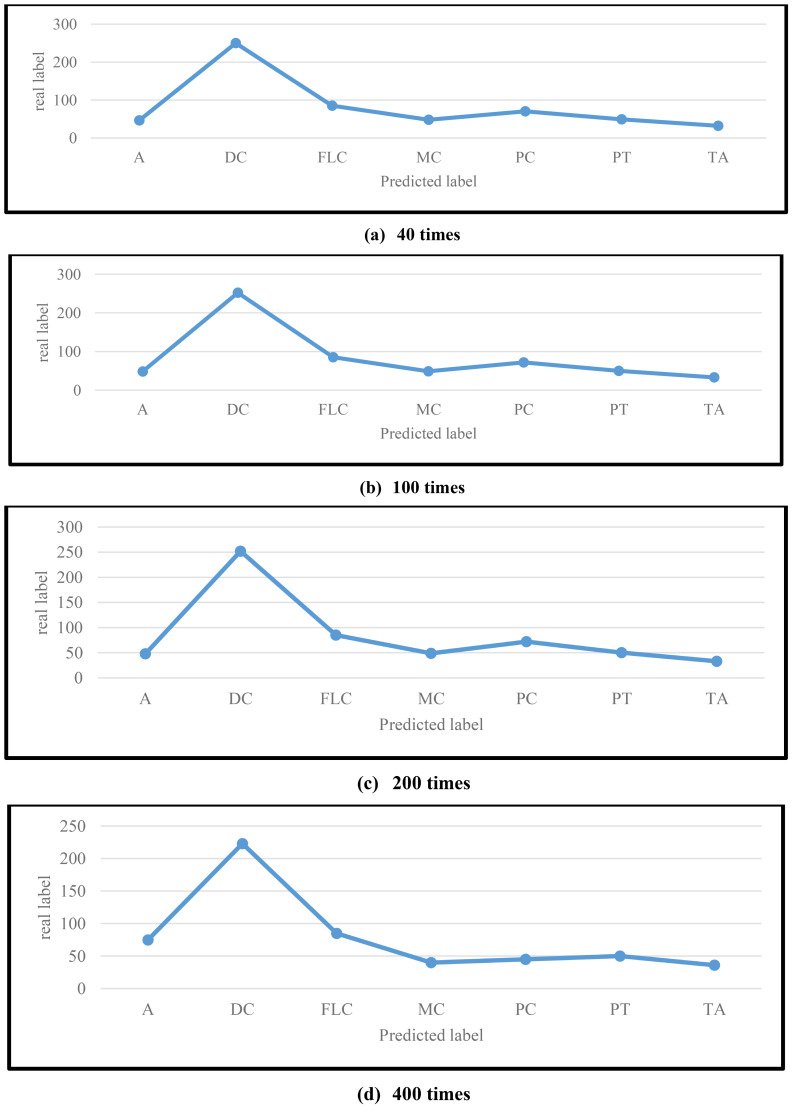
Confusion matrices of the MVT-OFML model at different magnification levels for the 8-class task on the BreakHis dataset.

Compared with the best baseline model BreaST-Net, MVT-OFML achieves more significant performance improvements at any magnification level. For example, at 400x magnification, an **8.31%** improvement in accuracy can be observed, so that the categorization model is more useful in practice. Above all else, at 200x magnification, the MVT-OFML model improved the F1-score by 5.73 percent. It should be noted that when compared to other magnification levels, 400x yields the best performance benefits. The MVT-OFML model can handle high magnification well due to two key factors:

First, the explainable AI multi-view Transformer encoder can extract more robust and effective features independent of magnification, learning valuable information from other pathological categories to handle complex multi-classification tasks and achieve better performance; Second, the MVT-OFML model jointly mines complementary information between heterogeneous CNN and Transformer models by deeply utilizing the logits layer and intermediate feature layers. This simulates a real pathological diagnosis scenario, fully leveraging the advantages of CNN and Transformer models in their respective feature learning to extract more discriminative features from breast cancer pathological images. When pathological images are divided into instances and grouped into bags, Multiple Instance Learning (MIL) reduces the model’s reliance on annotations by converting the pathology image classification issue into a weakly supervised MIL problem. In pathological photos of breast cancer, the intricate textures, structures, and spatial information are not sufficiently captured by the conventional Transformer network. In order to obtain more pertinent pathological information from related pathological categories, the distillation temperature now more successfully softens the probabilities. Consequently, this enhances the precision of classifying breast cancer pathology images. Compared to the binary classification task, the 8-class classification of breast cancer is more challenging. Therefore, excellent performance on a more challenging task can better demonstrate the effectiveness and robustness of the model. The dual-stream network combining CNN and Transformer can capture more valuable deep pathological features. **Figure 5** shows the confusion matrix of the 8-class classification task on the BreakHis dataset by the proposed method.

#### 4.3.2 The BACH dataset: experimental findings

Compared with the BreakHis dataset, the BACH dataset contains images with higher resolution and more complex content, including stronger adhesion and more noise from the background, making classification on this dataset more challenging. Therefore, this section compares classification performance on the BACH dataset, and the detailed quantitative results are shown in **Table 5**. The MVT-OFML model is evaluated against several established benchmarks, including Patch+Vote (30), HybridDNN (Hybrid Deep Neural Networks) (31), 3E-Net (32), TransMIL (22), MA-MIDN (16) (Multi-view Attention-guided Multiple Instance Detection Network), and MSMV-PFENet (Multi-Scale Multi-View Progressive Feature Encoding Network) (33).

**Table 5:** Comparison of the performance of the MVT-OFML model and other mainstream methods on the BACH dataset.

Backbone network or method	Year of publication	Accuracy	Precision	Recall rate	F1 score
Patch+Vote (24)	2019	85	86.77	81.91	84.23
Hybrid DNN (25)	2020	95.29	94.46	94.43	94.31
3E-Net (26)	2021	96.68	95.46	95.45	95.46
TransMIL (27)	2021	85.83	86.9	84.69	85.78
MA-MIDN (28)	2021	93.57	96.18	94.26	95.18
MSMV-PFENet (29)	2022	94.8	95.2	94.89	94.79
MVT-OFML	2023	98.94	98.56	98.48	98.67
Improvement		2.26↑	3.10↑	3.03↑	3.21↑

The symbol “↑”defines the improved values.

Experimental findings demonstrate that MVT-OFML surpasses all baselines across every metric. In comparison with the strongest competitor, MSMV-PFENet, it achieves gains of 2.26% in accuracy, 3.10% in precision, 3.03% in recall, and 3.21% in F1-score. The MVT-OFML model is also effective for more difficult pathological image datasets.

Although large-sized BACH breast cancer pathological images contain a large amount of noise, the multi-view Transformer encoding module with cross-view attention mechanism can accurately capture the global contextual information in the images and form a positive complementarity with the local information (such as cell morphology, texture, and color) captured by the CNN model. This suppresses noise interference, reduces the negative impact of adhesion, and achieves more robust and superior classification performance, indicating that the MVT-OFML model is efficient and robust.

In addition, the online fusion mutual learning mechanism also plays an important role. ResNet-50 and the multi-view Transformer encoder effectively extract more informative pathological features from diverse intermediate representations. The ensemble classifier then reintegrates the deep pathological insights from both backbone models into the fusion module. Consequently, the output logits and distilled features from the two networks jointly strengthen the fusion classifier’s discriminative capacity. Together, the backbone networks and the fusion classifier synergize to uncover sufficient knowledge for classifying breast cancer pathology images, thereby enhancing overall classification accuracy.

**Figure 6:**
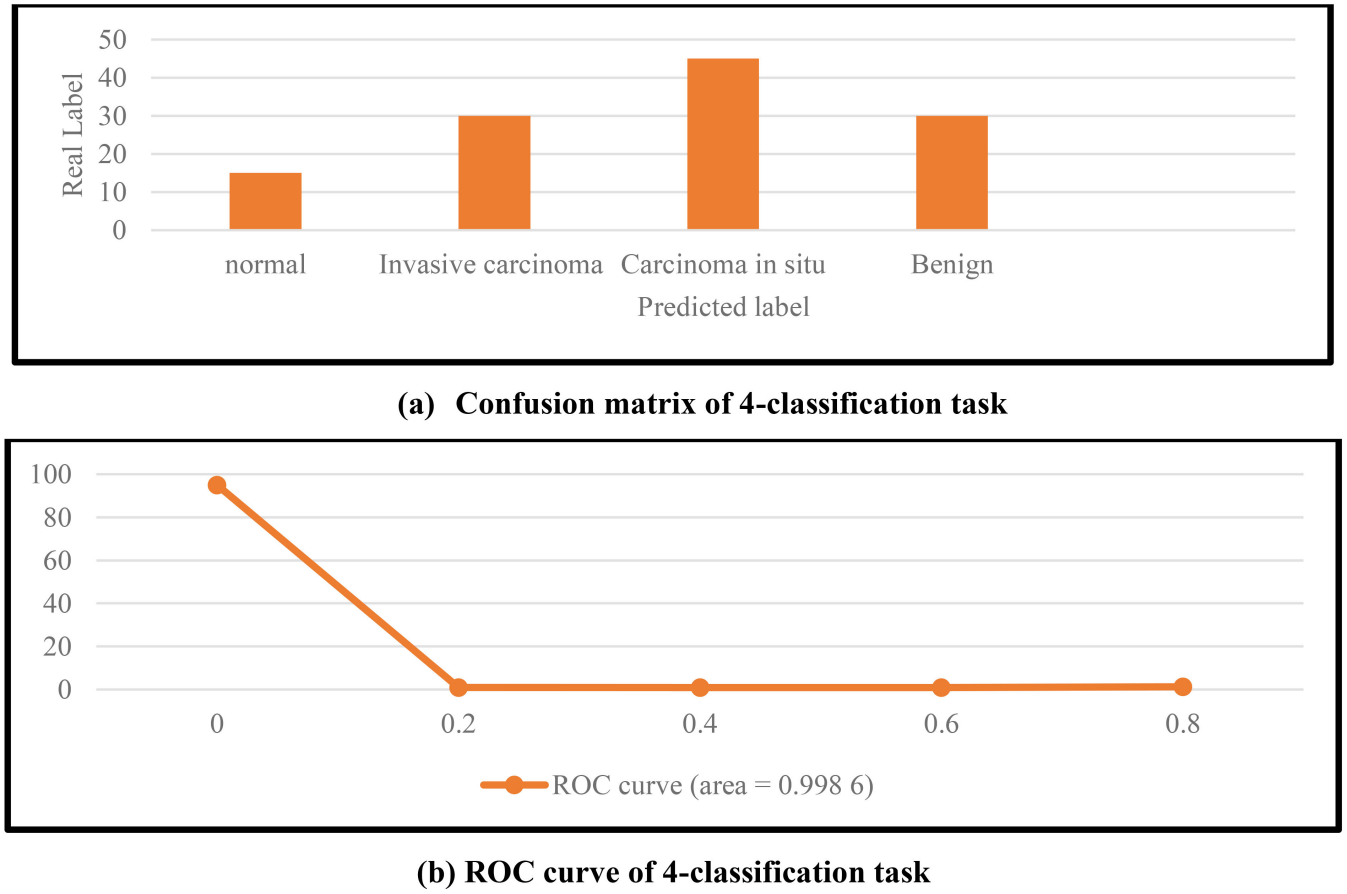
Results of the MVT-OFML model on the BACH dataset.

**Figure 6a** shows the classification confusion matrix of the MVT-OFML model on the BACH dataset. In **Figure 6a**, all four image types on the BACH dataset achieved good classification performance. The normal class images pose a greater classification challenge due to the small number of samples, and some samples were misclassified as invasive carcinoma. In the future, contrastive learning methods can be used to further enhance the discriminative power of features and reduce misclassification. **Figure 6b** shows the ROC curve of the MVT-OFML model. In **Figure 6b**, the MVT-OFML model achieved an AUC of 0.9976, indicating that the MVT-OFML model has excellent overall classification performance.

Based on all metrics, it can be concluded that the MVT-OFML model can learn key discriminative information from large-sized breast cancer pathological images, laying an important foundation for improving classification performance.

#### 4.3.3 Ablation study

To evaluate the actual contribution of each component of the MVT-OFML model, ablation experiments were conducted. The ablation experiments were completed on the 8-class classification task of the BreakHis dataset. Several variant models were designed, including: ResNet-50 (Model A), Transformer (Model B), ResNet-50 + Transformer (Model C), ResNet-50 + Transformer + Multi-view Encoding (Model D), ResNet-50 + Transformer + Multi-view Encoding + EC (Model E), ResNet-50 + Transformer + Multi-view Encoding + AFC (Model F), ResNet-50 + Transformer + Multi-view Encoding + EC + AFC + Mixup Data Augmentation (Model G), and the full MVT-OFML model. Here, “EC” denotes the Ensemble Classifier, and “AFC” denotes the Fusion Classifier. The experimental results are shown in **Table 6**.

**Table 6:** Ablation analysis experimental results on the BreakHis dataset.

Model	Mixup	ResNet-50	Transformer	EC	AFC	Accuracy	F1 score
A	×	✓	×	×	×	86.58	87.26
B	×	×	×	×	×	84.33	84.95
C	×	✓	×	×	×	88.75	88.69
D	×	✓	✓	×	×	93.56	94.07
E	×	✓	×	✓	×	94.33	94.34
F	×	✓	✓	×	✓	96.64	96.38
G	×	✓	✓	✓	✓	97.12	97
MVT-OFML	✓	✓	✓	✓	✓	98.65	98.83

As shown in **Table 6**, compared with ResNet-50 + Transformer (Model C), after adding the multi-view Transformer encoding module, the model accuracy increased significantly by 4.81%, indicating that the multi-view Transformer encoding is effective for breast cancer pathology image classification.

Based on Model D, adding the ensemble classifier EC (Model E) and adding the fusion classifier AFC (Model F) improved classification performance by 0.77% and 3.08%, respectively. This shows that the fusion classifier plays a more significant role. This is because the ensemble classifier, based on the idea of ensemble learning, only fuses the final results and does not process redundant data between heterogeneous features, so the performance improvement is relatively small. In contrast, after introducing the fusion classifier AFC, on the one hand, it adaptively fuses features from heterogeneous networks; on the other hand, by transferring pathological knowledge through FPKT (Fusion Pathological Knowledge Transfer), it fully utilizes the KL divergence to feed back the softened probability distribution of the fusion classifier to each sub-network. In heterogeneous networks, pathological knowledge describes circumstances in which false or misleading information spreads and affects decision-making and network performance. Suboptimal or even dangerous behaviors may result from contradictory or insufficient knowledge across various network elements. Collaboration among heterogeneous networks makes data and knowledge sharing essential. The propagation of “pathological knowledge,” which might impede network training and collaboration efforts, can result from this interaction if it is not carefully handled. The less redundant data between the heterogeneous network and the fusion classifier, the greater the KL divergence, and the fusion classifier can provide a stronger supervision signal to the heterogeneous network; conversely, the more redundant data the fusion classifier has on the heterogeneous network, the smaller the KL divergence, and the weaker the supervision signal the fusion classifier provides to the heterogeneous network. In this case, the cross-entropy loss between the logits output of the heterogeneous network and the true labels will provide a stronger supervision signal to the heterogeneous network. By jointly integrating the FPKT and the logits output of the heterogeneous network, the network parameters are dynamically adjusted, promoting network optimization and reducing the impact of redundant data on model training.

The fusion classifier contains deep pathological information from heterogeneous networks. Compared with AFC, the multi-view Transformer encoding module is more important because it can accurately capture the global contextual information of breast cancer pathological images and learn more valuable pathological knowledge from heterogeneous intermediate feature layers.

Finally, Mixup data augmentation also has a boosting effect on the model, with the greatest performance improvement coming from the MVT-OFML model, which combines multi-view Transformer encoding, ensemble classifier, and fusion classifier to enhance classification performance. Therefore, in the MVT-OFML model, the most critical component is the multi-view Transformer encoding module, followed by the fusion classifier, the ensemble classifier, and finally the Mixup data augmentation operation. The fusion classifier incorporates extensive pathological data from many networks. The multi-view Transformer encoding module is more significant than AFC since it can recognize more relevant pathological information from heterogeneous intermediate feature layers and precisely capture the global contextual information of breast cancer pathological images. The global contextual features present in tissue structures are captured by Transformer, while CNN concentrates on retrieving local data. Together, the global and local properties effectively illustrate pathological semantics in pictures. An ablation analysis was conducted on the specific intermediate features input to AFC to determine the optimal combination of intermediate features. The ablation experiments were completed on the 8-class classification task of the BreakHis dataset. The specific implementation is as follows: based on Model F (ResNet-50 + Transformer + Multi-view Encoding + AFC), ResNet-50 convolution layers 1 to 3 and Transformer layers 1 to 3 were respectively selected to construct combinations of intermediate features input to AFC, including:

AFC1 (ResNet-50 convolution layer 1 + Transformer layer 1), AFC2 (ResNet-50 convolution layer 2 + Transformer layer 2), and AFC3 (ResNet-50 convolution layer 3 + Transformer layer 3). The experimental results are shown in **Table 7**.

**Table 7:** Ablation analysis experimental results of specific middle layer features.

Model	Mixup	ResNet-50	Transformer	Multi-view encoding	EC	AFC	Accuracy	F_1_ Score
F	×	✓	✓	✓	×	AFC1	95.78	95.01
F	×	✓	✓	✓	×	AFC2	96.31	96.17
F	×	✓	✓	✓	×	AFC3	96.64	96.38

In **Table 7**, when the combination of ResNet-50 convolution layer 3 and Transformer layer 3 is used as intermediate feature input to the fusion classifier, the MVT-OFML model can achieve the best classification performance. Therefore, the MVT-OFML model performs online fusion mutual learning by combining features of ResNet-50 convolution layer 3 and Transformer layer 3 to achieve breast cancer pathology image classification.

#### 4.3.4 Feature visualization results

**Figure 7:**
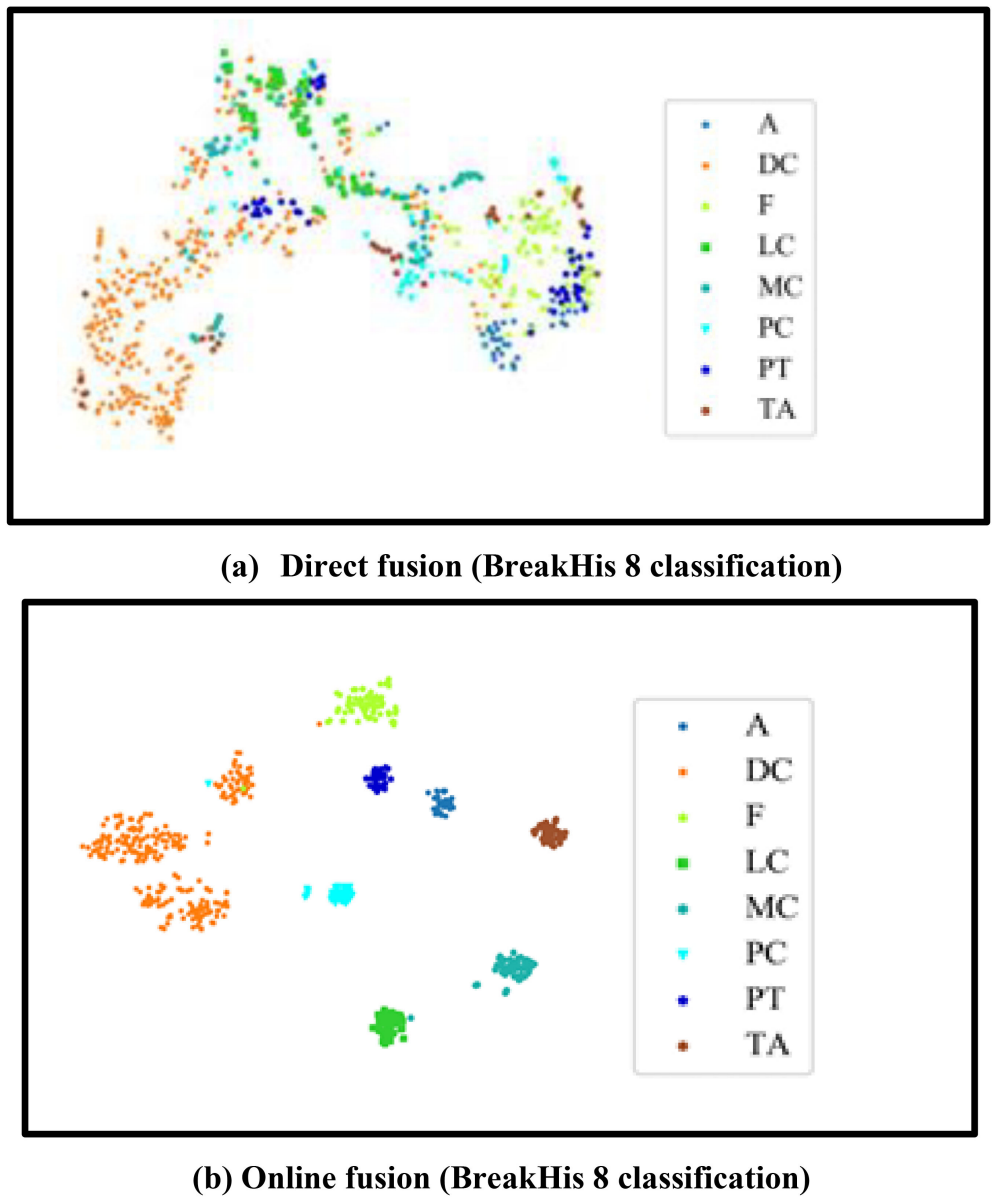
t-SNE visualization results.

In this section, t-SNE (34) is used to visualize the deep features generated by the model. The visualization experiments were conducted on the 8-class classification task of the BreakHis dataset, and the results are shown in **Figure 7**.

**Figure 7a** shows the visualization result of directly concatenating and fusing features at the last pooling layer of ResNet-50. **Figure 7b** shows the online fusion visualization result of the proposed MVTOFML method. Compared with direct fusion, the proposed model aggregates breast cancer pathological images of the same class together and pushes samples of different classes far apart, which helps construct a clear decision boundary and improve the classification accuracy of the model.

**Figure 8:**
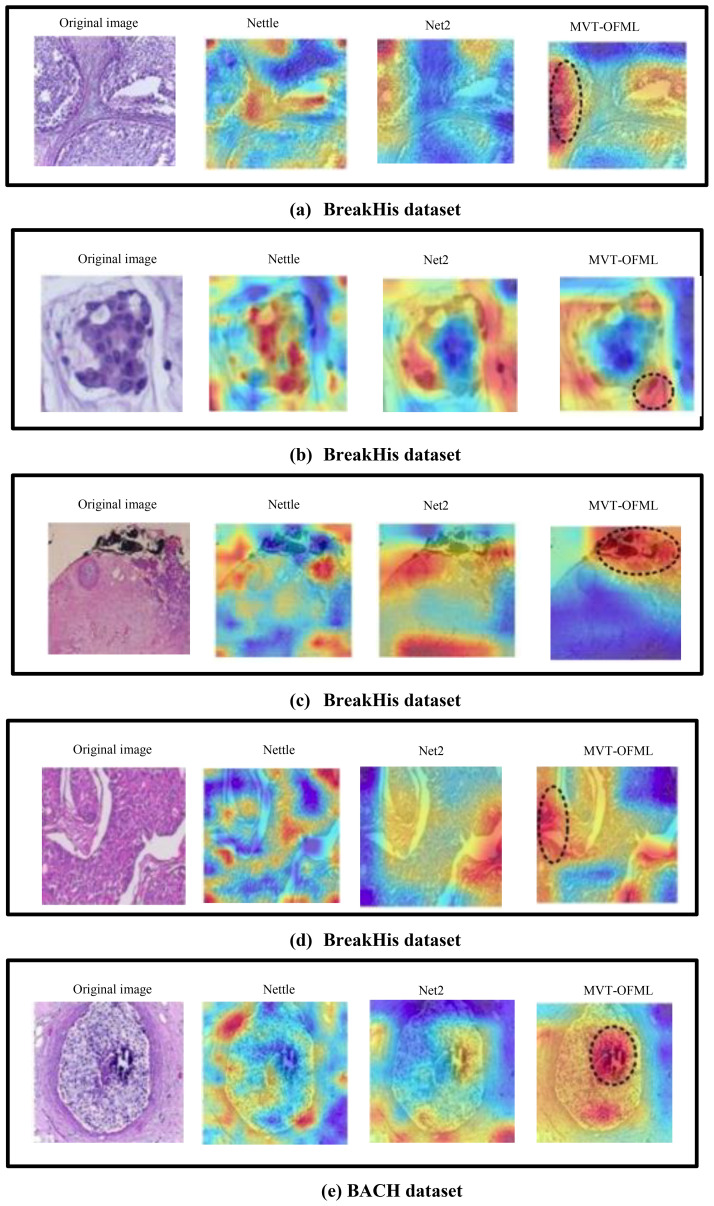
Grad-CAM visualization results.

Therefore, based on the visualization results, it can be concluded that the fused features produced by the MVTOFML model have stronger discriminative power, which is beneficial for improving the classification performance of breast cancer pathological images. Prior to completing classification, early studies preprocessed and segmented diseased pictures, then retrieved characteristics. MIL is frequently used for pathological diagnosis of Whole Slide pictures (WSI) and does well with high-resolution histopathology pictures. Breast cancer pathological picture classification uses both global contextual information about breast tissue structure and local nuclear characteristics. In this section, Grad-CAM (35) is used to visualize the areas the network focuses on. **Figure 8** compares the CAM visualization results obtained by ResNet-50, Transformer, and MVTOFML on the BreakHis and BACH datasets, as shown in **Figures 8a–e**. As shown in **Figure 8**, in the visualization images of ResNet-50, the model only focuses on fragmented local feature information. For example, in **Figures 8b, c**, ResNet-50 has limited localization ability for pathological images, and therefore cannot extract global information. To extract complementary information from logits, you must first understand that logits are the unnormalized, raw scores prior to being converted into probabilities using an activation function such as softmax. Considering the internal state of the model, such as the weights and biases used in the last layer to generate the logits, allows access to complementary information. Before the final activation function (such as sigmoid or softmax) is applied, a neural network’s output is called a logit. In the visualization images of the Transformer, although the model focuses on global contextual information, it cannot accurately localize local details, and some areas that do not need attention are also focused on — for example, **Figures 8c, d**. The MVTOFML model utilizes the rich information in the fused features to achieve localization of key lesion areas in breast cancer pathological images. In addition, the results in **Figure 8** possess a certain degree of interpretability, which can better assist doctors in clinical diagnosis activities. Ultrafast MRI with radiomics performs better than regular MRI in detecting invasive ductal carcinoma (IDC) of the breast. This method works well for identifying tiny tumors and distinguishing the molecular subtypes of breast cancer, including HER2 status. Furthermore, IDC identification using histopathology images has shown remarkable accuracy and sensitivity thanks to deep learning algorithms, especially Convolutional Neural Networks (CNNs).

## 5 Conclusion

Breast cancer pathology image classification not only relies on local nuclear features but also requires global contextual information of breast tissue structure. The MVTOFML model is proposed, which combines CNN and In addition, an online fusion mutual learning method is designed, which further improves breast pathology image classification performance by combining an ensemble classifier and a fusion classifier. Experiments show that the MVTOFML model outperforms mainstream methods on two public datasets. In the future, we plan to explore a multi-view bidirectional fusion mechanism to obtain richer and more discriminative pathological features.

## Data Availability

Publicly available datasets were analyzed in this study. This data can be found here: https://www.kaggle.com/datasets/ambarish/ break his https://www.kaggle.com/datasets/truthisneverlinear/bach-breast-cancer-histology-images.

## References

[1] Mastoi Q-u Latif S Brohi S Ahmad J Alqhatani A Alshehri MS. Explainable AI in medical imaging: An interpretable and collaborative federated learning model for brain tumor classification. Front Oncol. (2025) 15:1535478. doi: 10.3389/fonc.2025.1535478, PMID: 40083877 PMC11903279

[2] Rasool N Wani NA Bhat JI Saharan S Sharma VK Alsulami BS. CNN-TumorNet: Leveraging explainability in deep learning for precise brain tumor diagnosis on MRI images. Front Oncol. (2025) 15:1554559. doi: 10.3389/fonc.2025.1554559, PMID: 40206584 PMC11979982

[3] Smiley A Villarreal-Zegarra D Reategui-Rivera CM Escobar-Agreda S Finkelstein J. Methodological and reporting quality of machine learning studies on cancer diagnosis, treatment, and prognosis. Front Oncol. (2025) 15:1555247. doi: 10.3389/fonc.2025.1555247, PMID: 40297817 PMC12034563

[4] Hassan BAR Mohammed AH Hallit S Malaeb D Hosseini H. Exploring the role of artificial intelligence in chemotherapy development, cancer diagnosis, and treatment: present achievements and future outlook. Front Oncol. (2025) 15:1475893. doi: 10.3389/fonc.2025.1475893, PMID: 39990683 PMC11843581

[5] Wu D Li X. Prediction of two molecular subtypes of gastric cancer based on immune signature. Front Oncol. (2022) 12:826789. doi: 10.3389/fonc.2022.826789 PMC8802764 35111202

[6] Li X Zhu Y. MoGCN: A multi-omics integration method based on graph convolutional network for cancer subtype analysis. Front Oncol. (2022) 12:831676. doi: 10.3389/fonc.2022.831676, PMID: 35186034 PMC8847688

[7] Zhang J Zhao J. A novel model of tumor-infiltrating B lymphocyte specific RNA-binding protein-related genes with potential prognostic value and therapeutic targets in multiple myeloma. Front Oncol. (2021) 11:769456. doi: 10.3389/fonc.2021.769456, PMID: 34976013 PMC8719635

[8] Qu Y-Y Zhou Q. Pan-cancer analysis of the solute carrier family 39 genes in relation to oncogenic, immune infiltrating, and therapeutic targets. Front Oncol. (2021) 11:763842. doi: 10.3389/fonc.2021.763842, PMID: 34925450 PMC8675640

[9] Li L Cao J. Association of interleukin-10 polymorphism (rs1800896, rs1800871, and rs1800872) with breast cancer risk: An updated meta-analysis based on different ethnic groups. Front Oncol. (2022) 12:812345. doi: 10.3389/fonc.2022.812345, PMID: 35186043 PMC8855208

[10] Silva ITd Rosa JCD Zhao L. Advances in AI-based tools for personalized cancer diagnosis, prognosis and treatment. Front Oncol. (2021) 11:789123. doi: 10.3389/fonc.2021.789123

[11] Kapustina O Burmakina P Gubina N Serov N Vinogradov V. User-friendly and industry-integrated AI for medicinal chemists and pharmaceuticals. Artif Intell Chem. (2024) 2:100072. doi: 10.1016/j.aichem.2024.100072

[12] Chen X Peng Y. Counterfactual condition diffusion with continuous prior adaptive correction for anomaly detection in multimodal brain MRI. Expert Syst Appl. (2024) 254:124295. doi: 10.1016/j.eswa.2024.124295

[13] Pathology visions 2023 overview. J Pathol Inf. (2024) 15:100362. doi: 10.1016/j.jpi.2024.100362

[14] Seoni S Jahmunah V Salvi M Barua PD Molinari F Acharya UR. Application of uncertainty quantification to artificial intelligence in healthcare: A review of last decade (2013–2023). Comput Biol Med. (2023) 165:107441. doi: 10.1016/j.compbiomed.2023.107441, PMID: 37683529

[15] Pathology visions 2024 overview. J Pathol Inf. (2025) 16:100419. doi: 10.1016/j.jpi.2025.100419

[16] Kabir M Rahman A Hasan N Mridha MF. Computer vision algorithms in healthcare: Recent advancements and future challenges. Comput Biol Med. (2025) 185:109531. doi: 10.1016/j.compbiomed.2024.109531, PMID: 39675214

[17] Dai J Wang H Xu Y Chen X Tian R. Clinical application of AI-based PET images in oncological patients. Semin Cancer Biol. (2023) 91:124–42. doi: 10.1016/j.semcancer.2023.03.005, PMID: 36906112

[18] Erfanian N Heydari AA Feriz AM Iañez P Derakhshani A Ghasemigol M. Deep learning applications in single-cell genomics and transcriptomics data analysis. Biomedicine Pharmacotherapy. (2023) 165:115077. doi: 10.1016/j.biopha.2023.115077, PMID: 37393865

[19] Lu Y Wang A. Integrating language into medical visual recognition and reasoning: A survey. Med Image Anal. (2025) 102:103514. doi: 10.1016/j.media.2025.103514, PMID: 40023891

[20] Prelaj A Miskovic V Zanitti M Trovo F Genova C Viscardi G. Artificial intelligence for predictive biomarker discovery in immuno-oncology: a systematic review. Ann Oncol. (2024) 35:29–65. doi: 10.1016/j.annonc.2023.10.125, PMID: 37879443

[21] Holzinger A Dehmer M Emmert-Streib F Cucchiara R Augenstein I Ser JD. Information fusion as an integrative cross-cutting enabler to achieve robust, explainable, and trustworthy medical artificial intelligence. Inf Fusion. (2022) 79:263–78. doi: 10.1016/j.inffus.2021.10.007

[22] Shannon CP Lee AHY Tebbutt SJ Singh A. A commentary on multi-omics data integration in systems vaccinology. J Mol Biol. (2024) 436:168522. doi: 10.1016/j.jmb.2024.168522, PMID: 38458605

[23] Duan J Xiong J Li Y Ding W. Deep learning based multimodal biomedical data fusion: An overview and comparative review. Inf Fusion. (2024) 112:102536. doi: 10.1016/j.inffus.2024.102536

[24] Lin T Yu Z Xu Z Hu H Xu Y Chen C-W. SGCL: Spatial guided contrastive learning on whole-slide pathological images. Med Image Anal. (2023) 89:102845. doi: 10.1016/j.media.2023.102845, PMID: 37597317

[25] Nazir S Dickson DM Akram MU. Survey of explainable artificial intelligence techniques for biomedical imaging with deep neural networks. Comput Biol Med. (2023) 156:106668. doi: 10.1016/j.compbiomed.2023.106668, PMID: 36863192

[26] Islam T Hafiz S Jim JR Kabir M Mridha MF. A systematic review of deep learning data augmentation in medical imaging: Recent advances and future research directions. Healthcare Analytics. (2024) 5:100340. doi: 10.1016/j.health.2024.100340

[27] Shamshad F Khan S Zamir SW Khan MH Hayat M Khan FS. Chapter 4 - Transformer for medical image analysis. In: Zhou SK Greenspan H Shen D, editors. The MICCAI Society book Series, Deep Learning for Medical Image Analysis, 2nd ed. UNC-Chapel Hill, USA: Academic Press (2024). p. 99–131. doi: 10.1016/B978-0-32-385124-4.00012-X

[28] Parvaiz A Khalid MA Zafar R Ameer H Ali M Fraz MM. Vision Transformers in medical computer vision—A contemplative retrospection. Eng Appl Artif Intell. (2023) 122:106126. doi: 10.1016/j.engappai.2023.106126

[29] Cheng H Liu X Zhang J Dong X Ma X Zhang Y. GLMKD: Joint global and local mutual knowledge distillation for weakly supervised lesion segmentation in histopathology images. Expert Syst Appl. (2025) 279:127425. doi: 10.1016/j.eswa.2025.127425

[30] Tuly SR Ranjbari S Murat EA Arslanturk S. From Silos to Synthesis: A comprehensive review of domain adaptation strategies for multi-source data integration in healthcare. Comput Biol Med. (2025) 191:110108. doi: 10.1016/j.compbiomed.2025.110108, PMID: 40209575

[31] Singh SS Kumar S Ahuja R Barua J. Fusion of quantum computing and explainable AI: A comprehensive survey on transformative healthcare solutions. Inf Fusion. (2025) 122:103217. doi: 10.1016/j.inffus.2025.103217

[32] Zhao Z Liu Y Wu H Wang M Li Y Wang S. CLIP in medical imaging: A survey. Med Image Anal. (2025) 102:103551. doi: 10.1016/j.media.2025.103551, PMID: 40127590

[33] Jiang H Zhou Y Lin Y Chan RCK Liu J Chen H. Deep learning for computational cytology: A survey. Med Image Anal. (2023) 84:102691. doi: 10.1016/j.media.2022.102691, PMID: 36455333

[34] Jiang L Zhang C Zhang H Cao H. A lightweight spatially-aware classification model for breast cancer pathology images. Biocybernetics Biomed Eng. (2024) 44:586–608. doi: 10.1016/j.bbe.2024.08.011

[35] Jiang X Wang S Zhang Y. Vision transformer promotes cancer diagnosis: A comprehensive review. Expert Syst Appl. (2024) 252:124113. doi: 10.1016/j.eswa.2024.124113

